# Chasing the digital high: should hedonic consumption be viewed through the lens of behavioral addiction?

**DOI:** 10.3389/fpsyt.2025.1681307

**Published:** 2025-10-14

**Authors:** Shamini James, S. Karthik, Binu Thomas

**Affiliations:** ^1^ Kalasalingam Academy of Research and Education (Deemed to be University), Krishnan Kovil, India; ^2^ Amal Jyothi College of Engineering, Kottayam, India; ^3^ Marian College Kuttikkanam Autonomous, Kuttikkanam, India

**Keywords:** compulsive buying disorder, behavioral addiction, hedonic consumption, online shopping, digital psychiatry

## Introduction: digital desires and the rise of hedonic consumption

In today’s algorithm-based market, the way people buy things has changed. Instead of buying only what they need, many now make purchases based on their wants and emotions (1) Early research on compulsive buying highlighted its chronic and maladaptive nature long before the rise of digital platforms. Groundbreaking research by Faber and O’Guinn in the late 1980s established compulsive buying as a disorder with irresistible urges, financial damage, and emotional distress, setting the stage for future diagnostic controversies (2). Concurrent studies have established the consistency of these habits over time and contexts, further suggesting that compulsive buying is not situational but rather a stable behavioral phenotype. This trend is especially seen in Generation Z, who grew up with digital technology where fast satisfaction and highly personalized ads are common. In the past, shopping was mostly about simple transactions, but now it has become an emotional activity that gives temporary happiness, comfort, or a sense of identity (3). Online platforms are no longer just places to buy things—they are carefully designed spaces that catch people’s attention and influence their decisions.

Among Generation Z, online platforms have made impulsive buying more common, turning it into an emotional act rather than just a financial choice (4). This rise in hedonic consumption—where people buy things for pleasure, excitement, or emotional satisfaction—has brought new risks (5). These shopping habits are often influenced by both brain-based reward systems and digital techniques that encourage repeated behavior. In some ways, they are similar to addiction patterns seen in gambling (6). This article suggests that compulsive online shopping in Gen Z should be looked at more seriously from a mental health point of view, as it might be an emerging type of behavioral addiction with effects on the brain, mind, and society. To avoid conceptual overlap, we briefly clarify key constructs central to this article. Hedonic consumption refers to purchasing driven primarily by pleasure, novelty, or emotional gratification rather than functional need. Digital addiction describes technology-mediated behaviors, such as online shopping or gaming, that exhibit impaired control, salience, and persistence despite harmful consequences (7). Algorithmic pleasure architectures denote the intentional design of digital platforms that use personalization, persuasive cues, and behavioral data to amplify engagement and stimulate reward responses. These distinctions frame the subsequent discussion more precisely.

To maintain conceptual clarity, a few metaphorical terms used in this article are defined here. Digital high refers to the short-lived surge of pleasure or excitement reported during impulsive online purchases. Frictionless impulse loop describes platform features—such as one-click checkout or stored payment data—that reduce barriers to repeated buying. Emotional architecture can be thought of as a deliberate effort to design digital interfaces in a way that causes or controls user emotions. Finally, neurocognitive vulnerability is applied to the greater susceptibility of certain individuals, particularly the young people to reward-related impulses due to active brain building. These are the definitions that are applied to give uniformity and avoid any unnecessary language during the discussion.

## Algorithmic pleasure architectures and the engineered impulse

Hedonic consumption in online spaces is not accidental—it is carefully designed (8). Platforms like Instagram Shopping, TikTok Shop, and Amazon use real-time behavior tracking, machine learning, and targeted ads to understand and influence how users shop (9).These systems are designed to optimize user engagement through sensory appeal, gamified shopping experiences, and the presentation of “personalized” products based on browsing history and emotional data (10).

These platforms implement persuasive design techniques that are based on behavioral economics such as scarcity effect (Only 1 left)!, Urgency, social messaging (Buy now or miss out)! and proof (“Over 100 bought this today) to cause cognitive dissonance and expectation of rewards. (11). Such persuasive cues are thought to engage mesolimbic pathways, particularly dopaminergic circuits implicated in reward anticipation. While some early studies suggest that repeated exposure may contribute to neural adaptations resembling those seen in other compulsive behaviors, these possibilities remain provisional. Stronger evidence from longitudinal neuroimaging is needed before causal claims about neuroplastic change can be confirmed. The shopping experience becomes less about the utility of the item and more about the emotionally charged ritual of discovery, anticipation, and gratification (12).

## Neuroscience and the rewiring of reward circuits

From a neuroscience perspective, the parallels between online compulsive shopping and substance-related disorders are increasingly difficult to ignore (13). fMRI studies have shown that individuals with compulsive buying tendencies exhibit heightened activity in the nucleus accumbens and orbitofrontal cortex when exposed to shopping cues—regions implicated in the anticipation of reward and decision-making (14). Preliminary findings from neuroimaging suggest that chronic overstimulation of reward-related regions *may* alter sensitivity to natural rewards, potentially leading individuals to seek higher stimulation to achieve similar psychological effects. However, such interpretations are still tentative and require replication in larger, longitudinal samples (Kazmi et al., n.d.).

Emerging evidence points to a possible role for dopaminergic function in circuits regulating impulse control and emotional regulation. While this provides a plausible biological substrate for compulsive online shopping, current data remain insufficient to firmly establish dysfunction or causality (15). When combined with a developing prefrontal cortex—as is the case with adolescents and young adults—the capacity for risk assessment and delayed gratification diminishes (16). The result is a neurocognitive landscape particularly vulnerable to compulsive hedonic behaviors. This neural susceptibility is further exacerbated by the emotionally charged content embedded within digital shopping platforms (17) It is important to note that much of the neuroscientific evidence linking compulsive online shopping to reward-circuit dysregulation remains preliminary. While early neuroimaging and clinical studies point toward dopaminergic involvement and potential neuroplastic adaptations, these findings should be interpreted with caution. Larger-scale, longitudinal, and meta-analytic research is still needed to establish causal pathways. By framing current insights as emerging evidence rather than definitive conclusions, this article situates compulsive online shopping within a developing field of inquiry rather than a settled diagnostic reality.

## Emotional regulation and identity performance

For Generation Z, whose formative years have been spent in the dual spaces of real and virtual worlds, consumption has evolved into a form of identity performance (18). In an ecosystem where likes, shares, and brand affiliations construct social capital, the act of buying is deeply embedded in emotional expression and peer validation (19). Many Gen Z users report turning to online shopping not in response to material need, but to combat feelings of boredom, loneliness, anxiety, or sadness (20).

This aligns with the broader concept of hedonic adaptation, wherein individuals seek novel stimuli to maintain emotional equilibrium. In this context, online shopping provides an accessible and socially acceptable outlet (21). However, the compulsion to continuously seek out the next digital “high” may erode the individual’s capacity for delayed gratification, financial responsibility, and emotional resilience (22).

This emotion-driven consumption indicates that online shopping has taken on a mood-regulatory function. Like alcohol or binge eating in traditional addiction models, shopping becomes a way to displace psychological discomfort (23). What differentiates it in the digital era is the continuous accessibility and the frictionless nature of transactions—users can indulge in the behavior anytime, anywhere, without real-world constraints or social scrutiny (24). Understanding the emotional drivers of compulsive online shopping provides an important entry point into its deeper mechanisms. The same mood-regulatory tendencies that make shopping appealing are closely tied to neural processes that govern reward anticipation and impulse control. Moving from the psychological to the biological level, it becomes essential to examine how brain-based reward systems interact with digital environments. This connection creates a bridge between individual vulnerability and the structural designs of online platforms that amplify such behaviors. **Table 1** presents a conceptual framework illustrating how digital architectures, neurocognitive mechanisms, and emotional regulation processes interact to reinforce compulsive online shopping behaviors.

**Table 1:** Conceptual framework linking digital environments, neurocognitive processes, and compulsive online shopping.

Level	Key factors	Examples	Impact on compulsive shopping
Digital Architecture	Persuasive design, personalization algorithms, dark patterns	Infinite scrolling, one-click checkout, scarcity cues (“Only 1 left!”)	Reduces friction, amplifies urges, increases frequency of purchases
Neurocognitive Mechanisms	Reward pathways, dopaminergic activation, impulse control	fMRI evidence of nucleus accumbens activation; underdeveloped prefrontal cortex in youth	Heightened sensitivity to shopping cues, diminished capacity for delayed gratification
Emotional Regulation	Mood repair, identity construction, social validation	Shopping to reduce loneliness/anxiety; purchases linked to peer approval	Shopping becomes a mood-regulatory strategy, reinforcing the cycle
Behavioral Outcomes	Compulsive buying, financial harm, impaired control	Persistent shopping despite debt, distress, or conflict	Mirrors addiction-like “core components” (salience, loss of control, tolerance, withdrawal, persistence)

## Digital manipulation and ethical boundaries

These behavioral and psychological insights are exploited by digital architectures that are designed not only to satisfy desires but to manufacture them (25). The user interfaces of e-commerce platforms are constructed to bypass reflective cognition and nudge users toward impulsivity (26). A well-known example is the use of infinite scrolling, which eliminates decision points and enables extended engagement.

Similarly, the removal of checkout friction through saved payment information and biometric confirmation accelerates purchasing without conscious reflection. The platform becomes a perfectly tuned hedonic delivery system, calibrated through user feedback and algorithmic refinements (27).

Moreover, the data harvested from user behavior is used to create feedback loops that make future triggers even more effective. In effect, the user becomes both the target and the tool of behavioral prediction. These manipulative tactics are often hidden behind the veneer of personalization and convenience.

While digital design in consumer technology has outpaced regulation, psychiatric understanding of its impact remains underdeveloped. If we accept that certain patterns of online shopping behavior mirror those of established addictions, it becomes imperative to subject these environments to ethical scrutiny and regulatory oversight (28).

## Diagnostic considerations and psychiatric frameworks

Despite growing recognition of the compulsive nature of online consumption, mainstream diagnostic systems such as the DSM-5 or ICD-11 (29) have not yet formally recognized compulsive buying disorder or online shopping addiction as distinct clinical conditions. This is not due to a lack of evidence but rather the complexity in defining clinical thresholds and distinguishing pathological behavior from culturally sanctioned consumerism (30).

Proposed criteria for diagnosis often include preoccupation with shopping, repeated unsuccessful efforts to cut down, continued behavior despite harm, and emotional distress when prevented from shopping. These markers align closely with those used to diagnose behavioral addictions like gambling disorder (31). As summarized in **Table 2**, compulsive online shopping exhibits the core components of behavioral addictions, aligning closely with established disorders such as gambling and gaming.

**Table 2:** Comparison of core addiction components across behavioral addictions.

Core component	Compulsive online shopping	Gambling disorder	Gaming disorder
Salience (dominance of activity in thoughts/behavior)	Persistent preoccupation with online shopping, browsing products, or anticipating purchases	Preoccupation with betting outcomes, odds, or gambling activities	Preoccupation with gaming, planning sessions, or reviewing past games
Loss of Control	Repeated unsuccessful efforts to cut down or resist purchases	Inability to control gambling frequency or money spent	Inability to limit gaming time or disengage
Craving/Urges	Intense urges to shop triggered by cues (ads, notifications)	Strong urges to gamble in response to triggers	Compelling urges to play games despite other obligations
Tolerance (need for increasing engagement)	Requires frequent purchases or more expensive items to achieve the same satisfaction	Increasing amounts of money wagered for excitement	Longer gaming sessions or more stimulating games needed
Withdrawal-like Symptoms	Emotional distress, irritability, or anxiety when unable to shop	Restlessness or irritability when unable to gamble	Irritability, anxiety, or sadness when gaming is interrupted
Negative Consequences	Financial debt, academic/work impairment, interpersonal conflict	Financial loss, legal issues, relationship problems	Academic/work impairment, sleep disruption, social withdrawal
Continued Use Despite Harm	Persistent buying despite debt, conflict, or regret	Persistent gambling despite serious losses	Persistent gaming despite health, social, or occupational harm

A more inclusive diagnostic framework could place compulsive online shopping within the spectrum of behavioral addictions, possibly adjacent to gambling disorder. Such reclassification would allow for the development of targeted interventions, insurance reimbursement, and structured research funding (32).

## Diagnostic positioning and differentiation

While compulsive online shopping is not formally recognized in DSM-5-TR or ICD-11, (33) positioning it within existing nosological debates requires clear differentiation from related constructs. Impulse-control disorders (such as kleptomania or intermittent explosive disorder) are marked by episodic tension-relief cycles with poorly resisted urges, but without sustained salience or reinforcement (34). Coping-driven shopping—using purchases to manage loneliness, boredom, or sadness—is often normative unless it crosses thresholds of impaired control or functional harm. By contrast, behavioral addictions, as defined in ICD-11 for gambling and gaming, involve persistent engagement, loss of control, salience, escalation, and continuation despite negative consequences. Emerging evidence suggests compulsive online shopping demonstrates these addiction-like “core components”—including craving, failed cut-downs, tolerance, withdrawal-like distress, and impairment. Clarifying these boundaries strengthens the argument that compulsive online shopping should be considered along the behavioral-addiction spectrum, while still acknowledging ongoing nosological debates.

## Clinical interventions and preventive models

If compulsive online buying is to be seriously considered under the psychiatric lens, the clinical community must respond with strategies tailored to its unique digital features. Traditional models like Cognitive Behavioral Therapy (CBT) can be adapted to address compulsive shopping by focusing on cognitive distortions, emotional triggers, and behavioral cycles (35). Clients may benefit from thought records that analyze the impulse to purchase, mindfulness practices that enhance delay of gratification, and digital behavior diaries that track online engagement.

Motivational Interviewing (MI) is another promising approach, especially for ambivalent clients who may not perceive their buying behavior as problematic. Additionally, digital well-being programs and app-based interventions could be developed to promote shopping awareness, limit screen exposure, and provide supportive feedback (36). For young people, school-based awareness programs can help make conversations about impulsive shopping, social media influence, and emotional coping more acceptable (37). In more serious situations, where compulsive buying causes family problems or financial difficulties, family therapy might be necessary.

## Cross-cultural patterns and gendered experiences

From a cross-cultural point of view, compulsive shopping habits appear differently depending on cultural values, financial conditions, and access to digital technology (38). For example, in some East Asian countries, the strong focus on status symbols can increase the pressure to buy in order to fit in. On the other hand, in places facing economic hardship, people may turn to shopping as a way to mentally escape their struggles (39).

Gender differences also influence compulsive buying behavior. Women are more often diagnosed with this disorder, while men might not report it as much because of social stigma or because their habits show up differently, like collecting gadgets or gambling (40). These differences need to be considered during diagnosis and treatment. Mental health models should not generalize too much but instead include cultural and gender-sensitive approaches. It is important to understand how local traditions, global consumer trends, and personal weaknesses all interact to give proper psychological support (41).

Although the issue of compulsive online shopping is one of the most symbolic among the members of Generation Z, it should be noted that the generation is not a homogeneous group. The subpopulations are more vulnerable than others, with socioeconomic status, digital literacy, cultural norms, and parental influence being the determinants of the risk and resilience. As an example, greater financial literacy or good parental advice might act as protective factors, and economic disadvantage or low digital knowledge might lead to vulnerability. Recognizing these subtleties will make overgeneralization less likely and emphasize the necessity of more finely differentiated and context-aware studies.

## Policy, regulation, and public health imperatives

Legal and policy actions are very important to control the deeper problems that allow emotional buying to grow (17). Governments could make it necessary for online shopping platforms to follow ethical design rules, similar to the laws being discussed for children using social media. For example, stopping the use of dark patterns, limiting how often push notifications are sent, and adding features like time-outs or spending reports could help slow down or stop compulsive buying (42).

Moreover, collaborations between mental health professionals, consumer rights advocates, and technology developers can yield digital tools that are not merely user-friendly but also psychologically ethical (43). Digital literacy campaigns should teach users how algorithms work, fostering a sense of agency over one’s digital behavior (44). Universities and public institutions can play a pivotal role by incorporating these discussions into curricula for psychology, sociology, business, and media studies.

## Future research and global perspectives

Research must also catch up with lived realities. Longitudinal studies tracking the mental health impact of chronic impulsive buying among digital natives are urgently needed. Experimental studies exploring neural correlates and behavioral interventions will help solidify compulsive buying as a clinical construct (44). Furthermore, participatory research involving young people can capture the affective and symbolic meanings attached to consumption in the age of TikTok and virtual identities.

Public health discourse must evolve to include compulsive digital shopping as a potential threat to adolescent mental well-being. Integrating this recognition into national mental health strategies, digital governance frameworks, and education policy can create more holistic response (45). As digital environments continue to blur the boundaries between desire and disorder, psychiatry must remain flexible, interdisciplinary, and culturally responsive.

## Limitations and discussions

It is important to recognize opposing perspectives that caution against pathologizing all forms of online shopping. For many individuals, shopping functions as a normative form of leisure, self-expression, and even adaptive emotional regulation. Scholars have also emphasized consumer agency, suggesting that users are not merely passive recipients of algorithmic influence but retain meaningful decision-making capacity. From this standpoint, framing online shopping solely as “victimhood” may risk overlooking resilience and autonomy. Additionally, some degree of hedonic consumption can be adaptive, fostering identity exploration, social belonging, or stress relief. This article does not argue that all online shopping behaviors are pathological; rather, it highlights specific compulsive patterns that bear resemblance to behavioral addictions. Acknowledging these counterarguments strengthens the discussion by situating compulsive online shopping within an ongoing debate rather than a settled diagnostic category.

A further limitation of this article is that, while it critiques the manipulative tactics of digital platforms, it may have underexplored the precise thresholds where acceptable persuasion becomes coercion. To clarify, we suggest that persuasion remains ethical when it allows informed, reversible, and transparent choices, whereas coercion occurs when design features exploit cognitive vulnerabilities, obscure alternatives, or restrict user autonomy. Likewise, “digital harm” can be reasonably defined as the point where digital design contributes to impaired control, sustained distress, financial strain, or compulsive use despite adverse outcomes. Situating these thresholds within psychiatric criteria for behavioral addiction strengthens both the ethical and clinical implications of this discussion.

The evidence cited in this article spans neuroimaging studies, behavioral surveys, and clinical reports, each with inherent methodological limitations. Many neurobiological findings derive from small, cross-sectional samples with heterogeneous diagnostic criteria, which may reduce generalizability. Behavioral studies often rely on self-report measures that are vulnerable to bias. Cross-cultural and gender-based findings are similarly shaped by contextual differences in sampling and operational definitions. These considerations on quality imply that the existing findings could be discussed as tentative and hypothesis-forming, but not final. Future studies using standard diagnostic tools, longitudinal research design, and the evidence base will need more and bigger samples to be solidified.

## Conclusion

As the online space continues to develop into a psychologically compelling and algorithmically curated environment, it is essential that psychiatry rethink constructively the contours of hedonic consumption. What was once dismissed as impulsiveness or lifestyle choice now closely mirrors profiles of behavioral addiction—with neurobiological, affective, and social consequences, particularly among younger generations. Pathologizing compulsive online shopping as one type of lifestyle choice conceals the suffering and dysfunction it may obscure. Avowing such a phenomenon as a legitimate target for psychiatric scrutiny allows clinicians, researchers, and policymakers to open ethical, diagnostically informed, and therapeutically appropriate engagement commensurate with realities of living within one digitally enriched culture. Practically speaking, this point of view has three implications. In the case of psychiatry, it highlights the necessity to consider compulsive online shopping as a potential behavioral addiction, which should be given more diagnostic consideration and a place in the clinical discourse. In clinical practice, cognitive-behavioral therapy adapted, motivational interviewing, and youth-based awareness should be considered as some of the methods of tackling cognitive errors as well as emotional stimuli that drive the behavior. To curb manipulative design features which facilitate regulation and policy, structural protection can be enforced by encouraging ethical standards in platforms, and encouraging the digital literacy campaigns. This article aims to promote a multidimensional reaction sensitive to the vulnerability of the person and the digital ecosystems that define compulsive consumption.
